# A Minority Population of Non-dye-decolorizing *Bacillus subtilis* enhances the Azo Dye-decolorizing Activity of *Enterococcus faecalis*

**DOI:** 10.1264/jsme2.ME21080

**Published:** 2022-05-31

**Authors:** Yu Yamanashi, Tsukasa Ito

**Affiliations:** 1 Department of Environmental Engineering Science, Gunma University, Kiryu, Gunma, Japan

**Keywords:** amino acids, cross-feeding, metabolomics, minority, oxidation-reduction potential (ORP)

## Abstract

Microbes live in communities in biological wastewater treatment plants and in the intestines. However, limited information is currently available on the mechanisms by which minority bacterial populations assist other bacteria besides syntrophic relationships as well as on the microbial food web. Therefore, the present study investigated the effects of non-dye-decolorizing *Bacillus subtilis* strain S4ga at population levels ranging between 0.04 and 4% on the activity of dye-decolorizing *Enterococcus faecalis* strain T6a1 using a dye decolorization assay. The results obtained revealed that the minority population of *B. subtilis* S4ga enhanced the dye-decolorizing activity of *E. faecalis* T6a1, resulting in a shorter lag time and longer active time of dye decolorization. These effects were related to redox potential values rather than O_2_ concentrations. Comparisons of the extracellular metabolites in individual incubations of *E. faecalis* T6a1 and *B. subtilis* S4ga and a co-incubation suggested a mutual relationship through the cross-feeding of specific amino acids (tyrosine, methionine, tryptophan, phenylalanine, valine, and leucine from *B. subtilis* S4ga to *E. faecalis* T6a1; glutamine, histidine, aspartic acid, and proline from *E. faecalis* T6a1 to *B. subtilis* S4ga). An ana­lysis of intracellular primary metabolites indicated that the arginine deiminase (ADI) pathway, an ATP-producing energy-generating process, was more strongly activated in co-incubated *E. faecalis* T6a1 than in *E. faecalis* T6a1 incubated alone. These results suggest that a co-incubation with *B. subtilis* S4ga promoted ATP production by *E. faecalis* T6a1 cells and enhanced its dye-decolorizing activity.

*Enterococcus faecalis* has become an important bacterium. It has been shown to degrade azo dyes (Walker *et al.*, 1971; Liu *et al.*, 2007; Sahasrabudhe *et al.*, 2014), the main dyes present in textile wastewater (Ito *et al.*, 2018). Some strains are pathogens and nosocomial antibiotic-resistant bacteria known as vancomycin-resistant enterococci (Fisher and Phillips, 2009; Cassini *et al.*, 2019), whereas others produce the antimicrobial agent, bacteriocin, against spoilage bacteria (Foulquié Moreno *et al.*, 2006; Silva *et al.*, 2018) or are used as flavor enhancers in the food industry (Foulquié Moreno *et al.*, 2006). *E. faecalis* has also been suggested to function as a probiotic in humans and animals (Klein, 2003; Foulquié Moreno *et al.*, 2006). Furthermore, *E. faecalis* is a fecal indicator that is used for microbial source tracking in water environment management (Byappanahalli *et al.*, 2012). Since *E. faecalis* is present in communities with other bacteria under the above conditions, clarifying the mechanisms by which *E. faecalis* associates with other bacteria is the key to establishing strategies for its control.

Despite its low abundance in microbial communities, *Bacillus subtilis* exerts beneficial effects, such as the production of antimicrobial agents and surfactants (Ilinskaya *et al.*, 2017; Kaspar *et al.*, 2019), the attenuation of intestinal inflammation (Okamoto *et al.*, 2012; Zhang *et al.*, 2016; Ilinskaya *et al.*, 2017), and the modulation or restoration of the gut microbiota (Ma *et al.*, 2018; Hadieva *et al.*, 2021). Previous studies reported that *Bacillus* species in the wastewater treatment microbiota enhanced the removal of odors, organic compounds, and nitrogen by wastewater treatment systems (Murakami *et al.*, 1995; Park *et al.*, 2007; Ito *et al.*, 2019). The relative abundance of *Bacillus* species in wastewater treatment systems is low, ranging between 0.1 and 1% (Ito *et al.*, 2019). Nevertheless, *Bacillus* species have been suggested to cooperate with and enhance the activities of co-existing bacteria.

We have noticed from several literatures that *Bacillus* species often comprised the dye-decolorizing bacterial consortia, exhibiting higher decolorization efficiencies than individual bacteria (Itoh *et al.*, 2002; Patil *et al.*, 2010; Liu *et al.*, 2018). We investigated the dye-decolorizing bacterial community in river sediment polluted by textile wastewater (Ito *et al.*, 2016) and collected dye-decolorizing bacterial isolates (Ito *et al.*, 2018) to treat textile wastewater, which prompted us to create dye-decolorizing co-cultures. We coincidentally found that the dye-decolorizing activity of *E. faecalis* was enhanced when co-incubated with non-dye-decolorizing *B. subtilis*. Therefore, we focused on the combination of *B. subtilis* and *E. faecalis*. Even assuming an increase in *B. subtilis* in the activated sludge microbial community or in the gut microbial community, *B. subtilis* is only a part of these microbial communities. However, limited information is currently available on the effects of a low abundance of *B. subtilis* on major bacterial populations.

To investigate the role of the minor population of *B. subtilis*, its effects on the functional activity of the major population *E. faecalis* need to be assessed. Even though the dye decolorization assay is a low-tech method, it visualizes the activity of *E. faecalis* as a color change in test tubes within 1 day with high throughput. Green fluorescent protein (GFP) has been used to monitor *E. faecalis* in microbial communities (Haug *et al.*, 2010; Hoogenkamp *et al.*, 2015); however, GFP-generating fluorophores require oxygen (Cubitt *et al.*, 1995). The GFP-independent activity assay (dye decolorization assay) is suitable for targeting the dye-decolorizing enzyme azoreductase of *E. faecalis*, which is activated in reduced environments (Chung *et al.*, 1992). Another benefit of the dye decolorization assay using *E. faecalis* and *B. subtilis* is that each strain is easily culturable, distinguishable, and quantifiable on the same agar plate even though their population ratio is 100:1 or higher. We used a dye-decolorizing *E. faecalis* strain as the reporter strain and a non-dye-decolorizing *B. subtilis* strain as the partner strain with a minor population; when dye-decolorizing *E. faecalis* is activated, decolorization is enhanced.

In the present study, we evaluated the activities of *E. faecalis* when incubated alone and when co-incubated with *B. subtilis* using the dye decolorization assay. We also examined the abundance of non-dye-decolorizing *B. subtilis* needed to enhance the dye-decolorizing activity of *E. faecalis*. Furthermore, we investigated the mechanisms by which non-dye-decolorizing *B. subtilis* activated dye decolorization by *E. faecalis* using dissolved oxygen (DO) and the oxidation-reduction potential (ORP) and ana­lyses of intracellular and extracellular metabolites in the co-incubation and individual incubations of each strain.

## Materials and Methods

### Strains used in the present study and their identification

One bacterial strain (*E. faecalis* strain T6a1) exhibiting dye-decolorizing activity was isolated from the fingertips using a previously reported method (Ito, 2013; Ito *et al.*, 2018). Seven non-dye-decolorizing *Bacillus*-like strains (S4ga, S4gb, C16caA, C47ea, A14d3B, D3da, and C8ba) were isolated using the same method (Table S1). The full-length 16S rRNA gene sequences of these strains were analyzed using a colony polymerase chain reaction, followed by Sanger sequencing, which was performed at GENEWIZ Japan. The identification of these strains was based on an NCBI BLAST search for 16S rRNA gene sequences, colony morphology, and microscopy.

### Screening of non-dye-decolorizing *Bacillus* strains that enhanced dye decolorization by *E. faecalis* T6a1

Isolated strains were pre-incubated under two conditions: the incubation of each strain alone and a co-incubation of *E. faecalis* T6a1 with one of the seven *Bacillus* strains. Each strain was inoculated into 5‍ ‍mL of Luria-Bertani (LB) liquid medium in a test tube with a lid. After a static culture at 37°C for 17 h, 30‍ ‍μL of 10‍ ‍g‍ ‍L^–1^ Congo red dye (cat. no. 032-03922; Wako Pure Chemicals) was added to each test tube to achieve an initial dye concentration of 60‍ ‍mg‍ ‍L^–1^. After the addition of Congo red dye, samples were statically incubated at 37°C under oxygen-containing conditions by leaving the gas phase of the test tubes in the air. The color of the liquid in the test tubes was regularly observed. Dye concentrations were assessed by measuring the absorbance of the liquid in the test tubes at a wavelength of 490‍ ‍nm. To measure absorbance, liquid containing the cells and dye was centrifuged at 14,000×*g* for 5‍ ‍min, and the supernatant was analyzed using a spectrophotometer. The dye decolorization rate was calculated from the maximum slope of the line in each dye concentration–time graph. The dye decolorization assay was performed in triplicate.

Among the seven *Bacillus* strains examined, *B. subtilis* strain S4ga showed the highest acceleration of dye decolorization by *E. faecalis* T6a1, resulting in the complete decolorization of 60‍ ‍mg‍ ‍L^–1^ Congo red to 0‍ ‍mg L^–1^ (Table S1). An incubation of *E. faecalis* T6a1 alone decreased the dye concentration, but at a decolorization rate that was two-thirds that of the co-incubation with strain S4ga. Therefore, strain S4ga was selected for subsequent ana­lyses.

### Population ratio setting

To test different population ratios of *E. faecalis* T6a1 and *B. subtilis* S4ga, it was important to establish a stable community composition and quantify the population composition. We prepared pre-incubated *E. faecalis* T6a1 cells that were suspended in 10% LB medium with high turbidity and pre-incubated *B. subtilis* S4ga cells as follows. *E. faecalis* T6a1 and *B. subtilis* S4ga were separately pre-incubated in 100% LB medium at 37°C for 14 h. Cells were collected and washed with 10% LB medium, and each strain was then suspended in 10% LB medium. The cell turbidity of *E. faecalis* T6a1 was adjusted to approximately 3 O.D. in 10% LB medium and then mixed with pre-incubated *B. subtilis* S4ga. The population ratios of *B. subtilis* S4ga to *E. faecalis* T6a1 during the incubation were set to approximately 1% and also in the range of 0.04 to 40%. Population ratios were estimated based on cell turbidity after the pre-incubation and were confirmed by CFU counting. The cell number of *E. faecalis* T6a1 when incubated alone was adjusted to be similar to that of *E. faecalis* T6a1 in the co-incubation with *B. subtilis* S4ga.

The initial high cell turbidity of *E. faecalis* T6a1, which corresponded to approximately 7–9 log CFU mL^–1^, suppressed the growth of *E. faecalis* T6a1 and *B. subtilis* S4ga during the co-incubation. Although *B. subtilis* grows from 5 to 8‍ ‍log‍ ‍CFU‍ ‍mL^–1^ within 1 day when incubated alone, its growth was limited to 6‍ ‍CFU‍ ‍mL^–1^ when co-incubated with 8 log CFU mL^–1^ of *E. faecalis* in 10% LB medium. When the initial *E. faecalis* T6a1 population was 9 log CFU mL^–1^ and the initial *B. subtilis* S4ga population for the co-incubation was 7 log CFU mL^–1^, the populations of both strains did not significantly change before or after the incubation for 11 h (Fig. S1). Therefore, we controlled the population ratio of the strains during the co-incubation.

### Viable cell counts of *E. faecalis* T6a1 and *B. subtilis* S4ga

The amount of dye decolorized in the test tube was proportional to the population of dye-decolorizing *E. faecalis* T6a1. Therefore, *E. faecalis* T6a1 cell numbers were enumerated by dilution plating before and after the incubation. The incubated solutions collected from the incubation of *E. faecalis* T6a1 alone and the co-incubation of *E. faecalis* T6a1 and *B. subtilis* S4ga were spread on 10% LB agar medium with serial dilutions and then incubated at 37°C for 12 h, followed by colony counting. We previously reported a correlation between the colony counts of *B. subtilis* and sequence read frequencies by next-generation sequencing (Ito *et al.*, 2019). We confirmed that the colony count of *B. subtilis* correlated with the results of the sequencing ana­lysis at a quantification range of 0.001 to 1%. Although *B. subtilis* S4ga colonies appeared with *E. faecalis* T6a1 colonies on the same agar plate, they were easily discriminated based on their colony sizes (Fig. S2). *E. faecalis* T6a1 lacked motility and colonies were smaller (approximately 0.5‍ ‍mm in diameter), whereas *B. subtilis* S4ga was motile and had larger colonies (1.5–3‍ ‍mm in diameter) after an incubation for 12 h. *B. subtilis* S4ga colonies were further confirmed by their *Bacillus*-like wrinkled appearance after an incubation for 24–48 h. Some of the colonies were analyzed by colony-PCR and sequencing.

### Dye decolorization assay

Dye-decolorizing *E. faecalis* T6a1 was co-incubated with non-dye-decolorizing *B. subtilis* S4ga and Congo red dye at 37°C. *E. faecalis* T6a1 incubated alone was used as a control. Each strain was inoculated in 5‍ ‍mL of 10% LB liquid medium in a test tube with a lid. The dye decolorization assay was subsequently performed under oxic and anoxic conditions. Oxic conditions were created using air in the gas phase of the test tube. The initial DO concentration under oxic conditions ranged between 6 and 7‍ ‍mg‍ ‍L^–1^. In contrast, anoxic conditions were created by nitrogen purging of the gas phase in the tube for 60 s. After nitrogen purging, DO in the liquid in the test tube was less than 0.5‍ ‍mg‍ ‍L^–1^. The concentration of Congo red dye was set to 60‍ ‍mg‍ ‍L^–1^. Immediately after the addition of the dye, DO was routinely measured using the non-contact-type DO sensor, Fibox3-trace (PreSens Precision Sensing) until the liquid in the test tube was decolorized or the incubation was terminated. The ORP value of the liquid was measured when the incubation was terminated or when the dye in the liquid was completely decolorized. Initial ORP values ranged between +155 and +175 mV. pH did not significantly change before or after the incubation, ranging between pH 7.2 and 7.6. The viable cell count as CFU was evaluated after decolorization. The dye concentration during the incubation was assessed from the RGB of routinely acquired images (Fig. S3). The final dye concentration was evaluated by measuring absorbance at 490‍ ‍nm.

### Dye concentration estimations by measuring RGB values in images of incubating tubes

The time-course monitoring of dye concentrations was performed by measuring RGB values in the images acquired of test tubes during incubations. Standard solutions of Congo red dye were prepared in 10% LB medium at concentrations ranging between 0 and 300‍ ‍mg L^–1^ to obtain a calibration curve. Five milliliters of each standard solution was placed in a flat-bottomed test tube for a spectrophotometric ana­lysis (Spectroquant Empty cells 16‍ ‍mm; Merck KGaA), which was also used for incubations. Test tubes of standard solutions were placed in a photography box (WB-50; LPL) equipped with a LED backlight (SLT-B4C; MUTOH Industries) at an illuminance of 2,700 lx and images of test tubes were acquired using a camera with the following settings: shutter speed, 1/100 s; aperture, f/2.8; focal length, 4.5‍ ‍mm; ISO speed, 100 (Fig. S3A). After loading the images onto a PC, R, G, and B values at the center of each test tube were measured using the Eyedropper tool in PowerPoint (Microsoft). The total RGB value was calculated for each standard solution to create calibration curves of the total RGB value versus dye concentrations, yielding a linear approximation for concentrations <40‍ ‍mg L^–1^ and an exponential approximation for concentrations between 40 and 300‍ ‍mg L^–1^ (Fig. S3B). Regarding measurements of dye concentrations in incubation tubes, images of the test tubes were taken under the same conditions as those of the standard test tubes. Therefore, images were acquired under stable photography conditions. In addition, images of the tubes were taken along with those of the standard test tubes. We confirmed that the R, G, and B values of the standard test tubes were constant throughout the dye decolorization experiment. Furthermore, we evaluated dye concentrations in test tubes by a colorimetric assay using test tubes with the standard solutions. The final dye concentration was assessed by spectrophotometry. Therefore, we validated dye concentration estimations based on RGB measurements using multiple methods.

This method involves non-contact measurements that do not require the collection of liquid from test tubes and, thus, do not disturb the incubation solution in the test tube or the gas phase of the tube. Furthermore, these measurements may be performed quickly and repeatedly by acquiring images of test tubes.

### ORP measurements during the *B. subtilis* S4ga incubation

Some of the *B. subtilis* S4ga colony on the agar plate was suspended in 50‍ ‍mL of LB liquid medium and placed in an incubator at 37°C for 1 h. The cell suspension was centrifuged at 12,000‍ ‍rpm for 5‍ ‍min and the supernatant was replaced with 10% LB liquid medium. Centrifuged cells were re-suspended in 10% LB and, thus, a *B. subtilis* S4ga cell suspension (8 log CFU mL^–1^) was prepared in a 50-mL tube. In aerobic incubations, 200‍ ‍mL of 10% LB liquid medium was prepared in a 500-mL vial, and 240‍ ‍μL of the *B. subtilis* cell suspension was added to the medium. Congo red (10‍ ‍g‍ ‍L^–1^) was then added at a concentration of 60‍ ‍mg L^–1^. Three sets of vials were shaken at 60‍ ‍rpm. In microaerophilic incubations, 5‍ ‍mL of 10% LB liquid medium was prepared in a 16-mL test tube with a rubber stopper, and 30‍ ‍μL of the *B. subtilis* S4ga cell suspension was added to the medium. Congo red (10‍ ‍g‍ ‍L^–1^) was added at a final concentration of 60‍ ‍mg L^–1^. After the tube was capped with a rubber stopper, nitrogen gas was injected into the test tube with a needle for 3 s. DO was 1‍ ‍mg L^–1^ at 5 h and thereafter remained lower than 1‍ ‍mg L^–1^. During the incubation at 37°C, the ORP and DO of the incubation medium in the vials and test tubes were monitored for 27 h. Since it was necessary to open the caps of the vials and tubes to measure ORP, DO was assessed using a non-contact-type DO sensor before opening the vials. Unopened test tubes were opened each time to measure the ORP of the incubation solution under microaerophilic conditions. Therefore, 30 test tubes were initially prepared, and four to six test tubes were measured at each time point.

### Metabolomic ana­lysis

*(i) Incubation conditions.* To investigate whether *B. subtilis* S4ga affected the metabolism of *E. faecalis* T6a1, a metabolomic ana­lysis of the intracellular and extracellular water-soluble primary metabolites of strains T6a1 and S4ga was performed on co-incubated cultures and individual incubations of each strain. Incubations were conducted as described above (dye decolorization assay). Briefly, after a pre-incubation of each strain, the co-incubation of *E. faecalis* T6a1 and *B. subtilis* S4ga and individual incubations of each strain were performed at an initial Congo red dye concentration of 60‍ ‍mg L^–1^ in 10% LB medium at 37°C. Incubations were terminated at 10 h during decolorization. The use of 10% LB medium reduced background amino acids and vitamins derived from tryptone and the yeast extract of the incubation medium when intracellular and extracellular metabolites were extracted.

*(ii) Extraction and ana­lysis of intracellular water-soluble primary metabolites of *E. faecalis* T6a1.* To compare the intracellular metabolites of co-incubated *E. faecalis* T6a1 with those of *E. faecalis* T6a1 incubated alone, *B. subtilis* S4ga cells were removed from the co-incubation solution. To achieve this, we focused on cell sizes; *E. faecalis* T6a1 cells were spherical with a width of approximately 0.8‍ ‍μm and a length of 1.3‍ ‍μm, whereas *B. subtilis* S4ga cells were elongated with a width of approximately 0.8‍ ‍μm and a length of 2.6‍ ‍μm or longer (Fig. S4A and B). Therefore, we expected *B. subtilis* S4ga cells not to pass through a filter with a pore size of 0.8‍ ‍μm. Accordingly, a 0.8-μm filter was used to reduce the number of *B. subtilis* S4ga cells in the co-incubation solution. After a 10-h incubation with the dye, the co-incubation solution was filtered through a 0.8-μm membrane filter (DISMIC 25CS080AS; Advantec). The solution of *E. faecalis* T6a1 incubated alone was also filtered to ensure that the filtration procedure did not affect metabolites in *E. faecalis* T6a1 cells. Filtered solutions were filtered two more times to minimize contamination with *B. subtilis* S4ga cells. Although *B. subtilis* S4ga accounted for 2% of the total population after the 10-h incubation (Fig. S4C and D), the filtration treatment effectively removed vegetative cells of *B. subtilis* S4ga; none were observed among the approximately 10,000 cells of *E. faecalis* T6a1 examined by microscopy. We only detected some endospores or non-vegetative thin cells (Fig. S4E and F), accounting for 0.07% of the total population. This small population of non-vegetative cells did not affect the metabolomic ana­lysis of *E. faecalis* T6a1.

*E. faecalis* T6a1 cells prepared by filtration after the co-incubation and incubation alone were washed with phosphate-buffered saline once and then suspended in sterilized ultrapure water. The turbidity of the cell suspension of *E. faecalis* T6a1 with the co-incubation and in individual incubations was adjusted to be the same among all samples by the addition of ultrapure water. Methanol was then added to suspensions at a concentration of 80% to extract intracellular metabolites. 2-Morpholinoethanesulfonic acid (2-MES) and methionine sulfone were added as internal standards to cell suspensions at a final concentration of 10‍ ‍μM. After thorough mixing and centrifugation at 20,000×*g* for 5‍ ‍min, the supernatants of cell suspensions were collected for a metabolomic ana­lysis. Two sets of samples were prepared for the *E. faecalis* T6a1 co-incubation and individual incubations.

Ninety-seven water-soluble primary metabolites (*e.g.*, amino acids, organic acids, and coenzymes) were evaluated in intracellular extraction solutions of strain T6a1 by liquid chromatography tandem mass spectrometry (LCMS-8050; Shimadzu) equipped with a pentafluorophenylpropyl column. The resulting intensity was normalized to the height ratio (the peak height of each metabolite divided by the peak height of 2-MES). In cases in which a small peak was overlaid with the next peak or small peaks were considered to be noise, these peaks were regarded as undetected metabolites. Metabolic pathways in *E. faecalis* were analyzed using the Kyoto Encyclopedia of Genes and Genomes (KEGG) database (Kanehisa *et al.*, 2014).

*(iii) Extraction and ana­lysis of extracellular water-soluble primary metabolites from co-incubated cultures and *E. faecalis* T6a1 and *B. subtilis* S4ga incubated alone.* The incubation solutions of the co-incubated culture and *E. faecalis* T6a1 and *B. subtilis* S4ga incubated alone were centrifuged at 20,000×*g* for 7‍ ‍min, and the supernatants obtained were filtered using a 0.2-μm sterile membrane filter (ASFIL 033022SO-SFCA; AS ONE). In addition, 10% LB medium was used as a reference to measure the relative concentrations of amino acids from components of tryptone and the yeast extract (Zimbro *et al.*, 2009). Methanol was then added to filtered supernatants at a concentration of 80% to extract extracellular metabolites. 2-MES and methionine sulfone were added as internal standards to the supernatants at a final concentration of 10‍ ‍μM. Three sets of samples were prepared for co-incubated cultures and *E. faecalis* T6a1 or *B. subtilis* S4ga incubated alone, and 10% LB medium. Similar to the ana­lysis of intracellular metabolites, 97 water-soluble primary metabolites were evaluated in extracellular extraction solutions.

### Measurement of intracellular ATP of *E. faecalis* T6a1 and *B. subtilis* S4ga

The amount of intracellular ATP in the culture of *E. faecalis* T6a1 incubated alone was measured by subtracting the amount of extracellular ATP in the 0.2-μm filtration permeate from the total amount of ATP in the culture medium containing cells: ATP*_E.faecalis_*=ATP_total_–ATP_0.2‍ ‍μm-extracellular_. The amount of intracellular ATP in cultures of *B. subtilis* S4ga incubated alone was assessed in the same manner: ATP*_B.subtilis_*=ATP_total_–ATP_0.2‍ ‍μm-extracellular_. In the co-incubated culture of *E. faecalis* T6a1 and *B. subtilis* S4ga, the amount of intracellular ATP in *E. faecalis* T6a1 was measured by subtracting the amount of extracellular ATP in the 0.2-μm filtration permeate from the amount of ATP in the 0.8-μm filtration permeate containing extracellular ATP and *E. faecalis* T6a1 cells: ATP*_E.faecalis_*=(ATP_0.8‍ ‍μm-extracellular_+ATP_0.8‍ ‍μm-_*_E.faecalis_*)–ATP_0.2‍ ‍μm-extracellular_.

The amount of intracellular ATP in *B. subtilis* S4ga in the co-incubated culture was assessed by subtracting the amount of ATP in the 0.8-μm filtration permeate containing extracellular ATP and *E. faecalis* T6a1 cells from the total amount of ATP in the culture medium containing the extracellular and intracellular ATP of both strain cells: ATP*_B.subtilis_*=ATP_total_–(ATP_0.8‍ ‍μm-extracellular_+ATP_0.8‍ ‍μm-_*_E.faecalis_*). ATP measurements were performed using BacTiter Glo ATP measurement reagent (Promega) and a luminometer (Lu-mini; Viti Life Science Solutions) in accordance with the manufacturer’s instructions and the literature (Hironaka *et al.*, 2013).

### Statistical ana­lysis

The significance of differences was analyzed using two-tailed paired *t*-tests to compare the co-incubation with *B. subtilis* S4ga versus *E. faecalis* T6a1 incubated alone.

### Nucleotide sequence accession numbers

The 16S rRNA gene sequences of the isolated strains are available in the DDBJ/EMBL/GenBank databases under accession numbers LC557810–LC557817.

## Results and Discussion

### Effects of *B. subtilis* on the activity of *E. faecalis*

**Fig. 1.**
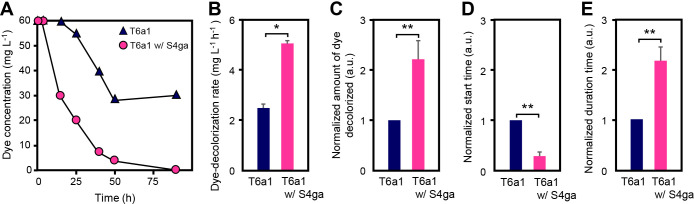
Effects of *Bacillus subtilis* S4ga on dye decolorization by *Enterococcus faecalis* T6a1. (A) Dye decolorization over time during the co-incubation of *B. subtilis* S4ga and *E. faecalis* T6a1 or by *E. faecalis* T6a1 incubated alone. (B) Dye decolorization rates of *E. faecalis* T6a1 incubated alone and co-incubated with *B. subtilis* S4ga for 11 h. (C) Ratio of the amount of dye decolorized by the co-incubation to the amount of dye decolorized by individual incubations. (D) Ratio of the time when the dye concentration started to decrease during the co-incubation to the time when the dye concentration started decreasing during the individual incubations. (E) Ratio of the time when the dye concentration continued to decrease with the co-incubation to the time when the dye concentration continued to decrease in individual incubations. Error bars represent standard deviations (B, C, D, and E); *P<0.05; **P<0.01; *n*=3 (B); *n*=9 (C, D, and E). a.u., arbitrary units.

We investigated the activity of *E. faecalis* T6a1 using the dye decolorization assay at a dye concentration of 60‍ ‍mg L^–1^. Fig. 1A shows typical changes in dye concentrations during the co-incubation of *E. faecalis* T6a1 and *B. subtilis* S4ga and *E. faecalis* T6a1 incubated alone. The *E. faecalis* T6a1 population during the co-incubation was approximately one-fifth of that when incubated alone at 48‍ ‍h: 1.8×10^8^ CFU mL^–1^ for the co-incubation and 8.4×10^8^‍ ‍CFU‍ ‍mL^–1^ when incubated alone. However, the dye concentration decreased earlier and faster during the co-incubation, and the amount of dye decolorized at 48‍ ‍h during the co-incubation was approximately two-fold that in individual incubations. In contrast, individual incubations did not show additional decolorization after 48 h. In the co-incubation, the population of *B. subtilis* S4ga was 1.4×10^5^‍ ‍CFU‍ ‍mL^–1^, and, thus, the population ratio of *B. subtilis* S4ga to *E. faecalis* T6a1 was 0.08%; this low ratio resulted in differences in the dye-decolorizing activity of *E. faecalis* T6a1. Furthermore, the maximum dye decolorization rate of the co-incubation was two-fold higher than that of individual incubations (Fig. 1B). We also repeatedly observed that the co-incubation doubled the amount of dye decolorized and halved the lag time to start decreasing the dye concentration from those in individual incubations (Fig. 1C and D). Additionally, the duration of dye decolorization was approximately 2.2-fold higher in the co-incubation than by *E. faecalis* T6a1 incubated alone (Fig. 1E). In other words, dye decolorization by the co-incubated strain started earlier and lasted longer than that of strains incubated alone, resulting in the decolorization of a large amount of dye. The population ratio of *B. subtilis* S4ga to *E. faecalis* T6a1 was 0.1%.

**Fig. 2.**
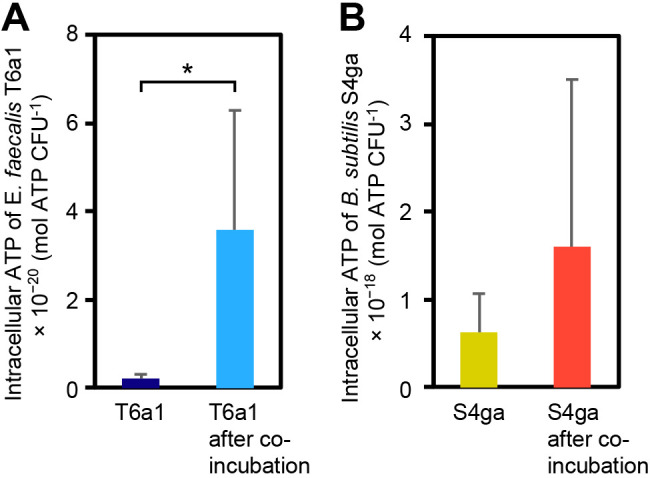
Intracellular ATP. (A) *Enterococcus faecalis* T6a1 incubated alone or co-incubated *E. faecalis* T6a1 after the removal of *Bacillus subtilis* S4ga at 24 h. (B) *B. subtilis* S4ga incubated alone or co-incubated *B. subtilis* S4ga after the removal of *E. faecalis* T6a1 at 24 h. Error bars represent standard deviations; *P<0.05; *n*=3 (A and B).

The co-incubation enhanced the decolorizing activity of *E. faecalis* T6a1. A shorter lag time of dye decolorization by *E. faecalis* T6a1 indicated that the co-incubation with *B. subtilis* S4ga affected the activity of *E. faecalis* T6a1 from the beginning of dye decolorization. Therefore, we measured the intracellular ATP levels of co-incubated *E. faecalis* T6a1 and that of *E. faecalis* T6a1 incubated alone. The intracellular ATP level of co-incubated *E. faecalis* T6a1 was 16-fold higher than that of *E. faecalis* T6a1 incubated alone (*P*=0.048, Fig. 2A). On the other hand, the intracellular ATP level of co-incubated *B. subtilis* S4ga was 2.5-fold higher than that of *B. subtilis* S4ga incubated alone (Fig. 2B). These results suggested that *E. faecalis* T6a1 and *B. subtilis* S4ga were both activated by the co-incubation. The difference in ATP values in Fig. 2B was not significant (*P*=0.44), which indicated that intracellular ATP levels varied more in co-incubated *B. subtilis* S4ga, even though the population of *B. subtilis* S4ga during the co-incubation was controlled within the same order.

### Population ratio of *B. subtilis* to *E. faecalis*

**Fig. 3.**
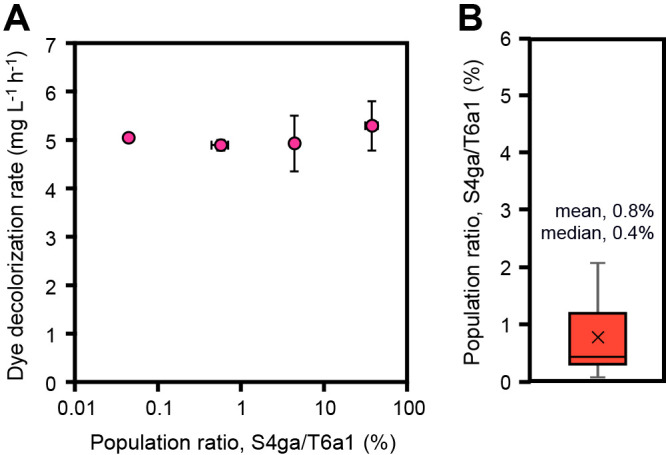
Effective population ratios of *Bacillus subtilis* S4ga to *Enterococcus faecalis* T6a1. (A) Dye decolorization rates of *E. faecalis* T6a1 co-incubated with *B. subtilis* S4ga at different population ratios ranging between 0.04 and 40%. (B) Distributions of the population ratios that enhanced the dye-decolorizing activity of *E. faecalis* T6a1. A box and whisker plot was created with data lower than 4% to focus on the effectiveness of the low ratio range. Error bars represent standard deviations; *n*=3 (A); *n*=8 (B).

We examined the effects of various population ratios of *B. subtilis* S4ga to *E. faecalis* T6a1 on the dye-decolorizing activity of *E. faecalis* T6a1 by changing the amount of *B. subtilis* S4ga added to the same amount of *E. faecalis* T6a1. Effective population ratios ranged between 0.04 and 40%. No significant differences were observed in the dye decolorization rates of the co-incubated cultures within the wide range of the *B. subtilis* S4ga population ratios (Fig. 3A). However, the dye decolorization rates of the co-incubations were higher than those of individual incubations at each time point (Fig. 1B and 3A). We created a box and whisker plot to show the distribution of the effective population ratio with data lower than 4% and demonstrated that 0.4–0.8% *B. subtilis *S4ga (less than 1%) enhanced the dye-decolorizing activity of *E. faecalis* T6a1 (Fig. 3B).

### Effects of *B. subtilis* on the oxidation state

**Fig. 4.**
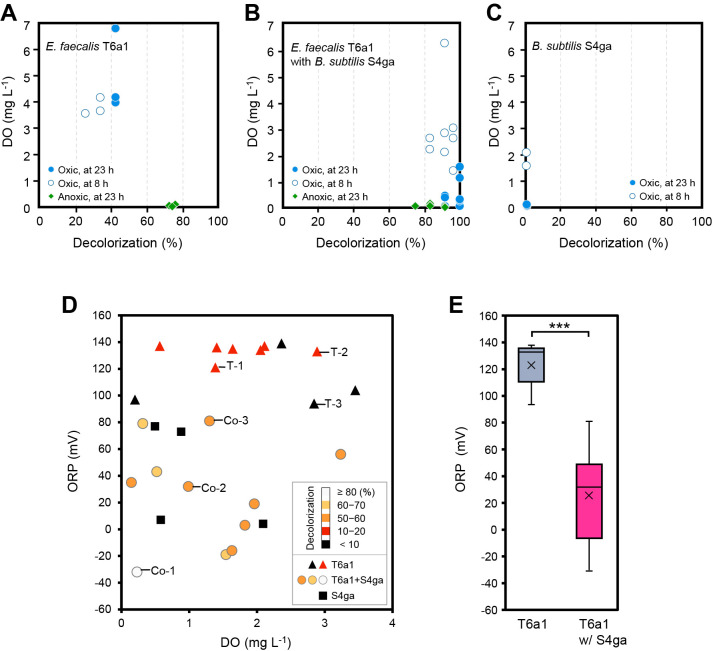
Impact of *Bacillus subtilis* S4ga on dissolved oxygen (DO) concentrations and the oxidation-reduction potential (ORP) during the co-incubation with *Enterococcus faecalis* T6a1 and during individual incubations with dye under oxic and anoxic conditions. Correlations between final DO and the dye decolorization rates of incubations of (A) *E. faecalis* T6a1 alone, (B) *E. faecalis* T6a1 with *B. subtilis* S4ga, and (C) *B. subtilis* S4ga alone at 8 and 23 h. (D) Correlation between final ORP and DO values after the incubation of *E. faecalis* T6a alone, *E. faecalis* T6a1 with *B. subtilis* S4ga, and *B. subtilis* S4ga alone for 20 h. Populations of *E. faecalis* T6a1 and *B. subtilis* S4ga at 20 h (CFU mL^–1^), Co-1: T6a1, 2×10^7^; S4ga, 7×10^5^ (S4ga-to-T6a1 population ratio, 3%). Co-2: T6a1, 2×10^8^; S4ga, 1×10^6^ (S4ga-to-T6a1, 0.6%). Co-3: T6a1, 7×10^7^; S4ga, 5×10^5^ (S4ga-to-T6a1, 0.7%). T-1: T6a1, 3×10^6^. T-2: T6a1, 2×10^7^. T-3: T6a1, 3×10^7^. (E) A box and whisker plot showing ORP values after the incubation of *E. faecalis* T6a1 alone or *E. faecalis* T6a1 with *B. subtilis* S4ga. ***P<0.001; *n*=11 (T6a1), *n*=11 (T6a1 with S4ga). Initial DO under oxic conditions ranged between 6 and 7‍ ‍mg L^–1^ (A, B, C, D, and E). The initial concentration of Congo red dye was 60‍ ‍mg L^–1^ (A, B, C, D, and E).

The dye-decolorizing enzyme azoreductase, which is expressed by *E. faecalis*, is activated in reduced environments (Chung *et al.*, 1992). Therefore, we investigated DO and the dye decolorization rates of co-incubated cultures of *E. faecalis* T6a1 and *B. subtilis* S4ga under oxic and anoxic conditions for 23 h (Fig. 4). The incubation of each strain alone was performed for comparison. The incubation of *E. faecalis* T6a1 alone achieved approximately 75% decolorization under anoxic conditions at 23 h, whereas the incubation of *E. faecalis* T6a1 alone showed a markedly lower decolorization rate (20–45%) under oxic conditions at 23 h, mainly between 3.5 and 4.2‍ ‍mg L^–1^ DO (Fig. 4A). On the other hand, the co-incubation of *E. faecalis* T6a1 and *B. subtilis* S4ga resulted in higher dye decolorization rates of 80–95% at 8 h under oxic conditions, mainly between 1.5 and 3.2‍ ‍mg L^–1^ DO, with further increases to 90–100% being observed at 23 h. Under anoxic conditions, the co-incubation showed incomplete dye decolorization of approximately 75–90% at 23 h (Fig. 4B). Under oxic conditions, the co-incubation of *E. faecalis* T6a1 and *B. subtilis* S4ga reduced DO to <3‍ ‍mg L^–1^, whereas the incubation of *E. faecalis* T6a1 alone did not.

We then examined the effects of *B. subtilis* S4ga on ORP values for dye decolorization by *E. faecalis* T6a1; *E. faecalis* T6a1 was incubated alone and co-incubated with *B. subtilis* S4ga under oxic conditions at < approximately 3‍ ‍mg L^–1^ DO (Fig. 4D). The initial ORP value was +163±11 mV in the presence of 1.9‍ ‍mg L^–1^ DO. The ORP values of the incubation of *E. faecalis* T6a1 alone ranged between +90 and +140 mV at 20 h. In contrast, the ORP values of the co-incubation ranged between –30 and +80 mV (Fig. 4E).

**Fig. 5.**
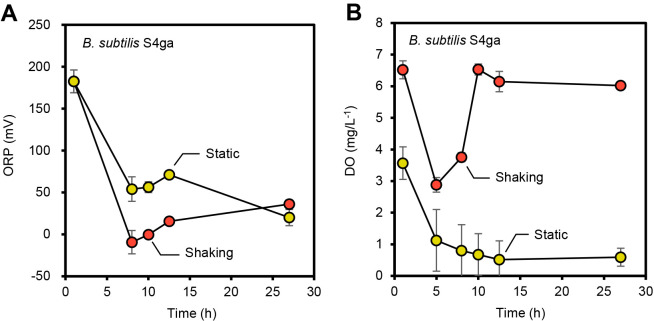
Effects of *Bacillus subtilis* S4ga on the oxidation-reduction potential (ORP) under oxic and microaerophilic conditions. Changes in ORP (A) and dissolved oxygen (DO) concentrations (B) over time during the incubation of *B. subtilis* S4ga alone with shaking and under static conditions. Rotational shaking at 60‍ ‍rpm created oxic conditions. A static incubation after nitrogen purging for 9‍ ‍s created microaerophilic conditions. The population of *B. subtilis* S4ga at 14 h: oxic, 1×10^8^ (CFU mL^–1^); microaerophilic, 0.5×10^8^ (CFU mL^–1^). The initial *B. subtilis* S4ga population was 3×10^5^ (CFU mL^–1^). Error bars represent standard deviations; *n*=3.

To confirm the reducing effects of *B. subtilis* S4ga on ORP, we monitored changes in ORP in *B. subtilis* S4ga incubated alone under oxic and microaerophilic conditions (Fig. 5). Under oxic conditions, *B. subtilis* S4ga reduced ORP to approximately 0 mV after an 8-h incubation after decreasing DO to <3‍ ‍mg L^–1^. Under microaerophilic conditions, *B. subtilis* S4ga reduced ORP to approximately +60 mV after an 8-h incubation with decreases in DO to <1‍ ‍mg L^–1^. *B. subtilis* S4ga created a moderately reduced environment when DO was present, and ORP levels were lower under oxic conditions than under microaerophilic conditions. These results demonstrated that *B. subtilis* S4ga reduced ORP to a level that enhanced dye decolorization by *E. faecalis* T6a1 under oxic conditions and also that oxygen was required to enhance dye decolorization by *E. faecalis* T6a1 when co-incubated with *B. subtilis* S4ga. *B. subtilis* has been widely used to produce amino acids (Su *et al.*, 2020) and vitamins (Abbas and Sibirny, 2011), and since oxygen is required for the production of these compounds (Utagawa, 2004; Hu *et al.*, 2017), *B. subtilis* may have produced reducing agents under oxic conditions, resulting in the lower ORP values observed in the present study.

### Extracellular metabolites of the co-incubation and individual incubations

**Fig. 6.**
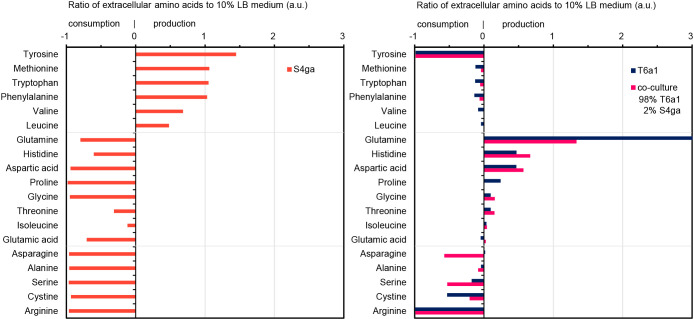
Extracellular amino acids produced or consumed by *Bacillus subtilis* S4ga incubated alone, *Enterococcus faecalis* T6a1 alone, and in a co-incubated culture of *E. faecalis* T6a1 and *B. subtilis* S4ga. Data represent the ratios of amino acids in the supernatants of incubated cultures at 14 h to amino acids in 10% LB medium at 0 h. Co-incubated culture: *E. faecalis* T6a1, 5×10^9^ (CFU mL^–1^); *B. subtilis* S4ga, 1×10^8^ (CFU mL^–1^) (S4ga-to-T6a1 population ratio, 2%). *n*=3. a.u., arbitrary units.

Extracellular metabolites in individual incubations of *E. faecalis* T6a1 and *B. subtilis* S4ga and the co-incubated culture revealed the characteristics of amino acid production and consumption from 10% LB medium (Fig. 6). The amino acids produced by *B. subtilis* S4ga were tyrosine, methionine, tryptophan, phenylalanine, valine, and leucine (*P*<0.05). *E. faecalis* T61a incubated alone utilized all these amino acids. However, decreases in the amounts of methionine, tryptophan, phenylalanine, valine, and leucine in the co-incubated culture were not as large as those with *E. faecalis* T6a1 incubated alone, which may be because *B. subtilis* S4ga provided the co-incubated culture with these amino acids. Although *B. subtilis* S4ga incubated alone produced a two-fold higher amount of tyrosine than that in 10% LB medium, the co-incubation of *E. faecalis* T6a1 with 1% *B. subtilis* S4ga consumed tyrosine and arginine, both of which may be rate-limiting substrates. The amino acids produced by *B. subtilis* S4ga (methionine, tryptophan, phenylalanine, valine, and leucine) are hydrophobic. Since hydrophobic amino acids are more permeable through the cell membrane than hydrophilic amino acids, *E. faecalis* T6a1 may readily acquire these amino acids. The amino acids produced by *E. faecalis* T6a1 were glutamine, histidine, aspartic acid, and proline (*P*<0.05). *B. subtilis* S4ga incubated alone utilized all these amino acids. The production and utilization of the amino acids produced by both strains demonstrated the cross-feeding of amino acids between *E. faecalis* T6a1 and *B. subtilis* S4ga, indicating synergistic effects on the activities of each strain.

### Intracellular metabolites of *E. faecalis* incubated with *B. subtilis*

**Fig. 7.**
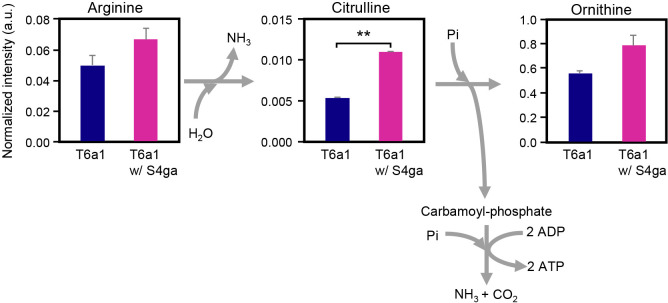
Metabolites of the arginine deaminase (ADI) pathway produced by *Enterococcus faecalis* T6a1 during the co-incubation with *Bacillus subtilis* S4ga and dye decolorization. Error bars represent standard deviations; **P<0.01; *n*=2. Data represent the normalized intensities of metabolites. a.u., arbitrary unit.

Among the 97 water-soluble primary metabolites examined, citrulline was the most significantly altered intracellular metabolite, with higher levels in *E. faecalis* T6a1 cells co-incubated with *B. subtilis* S4ga than in *E. faecalis* T6a1 cells incubated alone (Table S2). Citrulline is an amino acid produced during arginine degradation by the arginine deiminase (ADI) pathway in *E. faecalis* (Cunin *et al.*, 1986). The ADI pathway is an ATP-producing energy-generating process in *E. faecalis* (Slade and Slamp, 1952; Simon *et al.*, 1982; Barcelona-Andrés *et al.*, 2002). The ADI pathway catalyzes the conversion of arginine to citrulline and ammonia and further to ornithine, carbon dioxide, and ammonia, with the generation of 2 mol ATP mol^–1^ arginine (EC 3.5.3.6, EC 6.3.4.16). We demonstrated that ATP levels were more than 10-fold higher in co-incubated *E. faecalis* T6a1 cells than in *E. faecalis* T6a1 cells incubated alone (Fig. 2A). Although low oxygen tension is required to induce the ADI pathway in *Pseudomonas aeruginosa* (Lu *et al.*, 1999), oxygen tension in *E. faecalis* only weakly affects ADI pathway enzymes, and arginine is a more important inducer (Simon *et al.*, 1982; Barcelona-Andrés *et al.*, 2002). Similarly, higher dye-decolorizing activities by *E. faecalis* T6a1 were observed under oxic conditions (Fig. 4B), and extracellular arginine was extensively consumed by both *E. faecalis* T6a1 incubated alone and co-incubated *E. faecalis* T6a1 (Fig. 6). In addition, among the intracellular metabolites of *E. faecalis* T6a1, the detected amount of arginine was higher with the co-incubation than with *E. faecalis* T6a1 incubated alone (Fig. 7 and Table S2). Together with citrulline and arginine, the increase noted in ornithine was explained by the ADI pathway excreting an abundance of ornithine (Cunin *et al.*, 1986). Accordingly, we concluded that the co-incubation with *B. subtilis* S4ga activated the ADI pathway and promoted energy generation by *E. faecalis* T6a1 cells.

Proline levels were also significantly higher in co-incubated *E. faecalis* T6a1 cells. Proline is an abundant amino acid in *E. faecalis* enterolysin A (Nilsen *et al.*, 2003), which breaks down bacterial cell walls. Since some strains of *Bacillus*, including *B. subtilis*, show weak sensitivity to enterolysin A (Nilsen *et al.*, 2003), the presence of *B. subtilis* S4ga may stimulate enterolysin A production by *E. faecalis* T6a1. Moreover, we assumed that the presence of *B. subtilis* S4ga affected the oxidative stress response of *E. faecalis* T6a1 and focused on metabolites related to oxidative stress responses, such as glutathione and oxidized glutathione (Patel *et al.*, 1998; Portela *et al.*, 2014). The amount of glutathione detected in *E. faecalis* T6a1 incubated alone appeared to be slightly higher than that with the co-incubation. Further studies are needed to clarify whether oxidative stress in *E. faecalis* T6a1 is decreased by the co-incubation with *B. subtilis* S4ga.

The experimental conditions employed in the present study to analyze the intracellular primary metabolites of co-incubated *E. faecalis* T6a1 significantly minimized the contamination of metabolites from co-incubated *B. subtilis* S4ga. After the filtration treatment of the co-incubated culture, the *B. subtilis* S4ga cells observed by microscopy were non-vegetative cells, accounting for 0.07% of the total population. Based on our current knowledge of non-vegetative cells, they are not inactive, but are also not as active as vegetative cells. Therefore, the remaining non-vegetative cells in this small population did not appear to affect the ana­lysis of the intracellular primary metabolites of *E. faecalis* T6a1. In addition, assuming that the remaining *B. subtilis* S4ga cells were vegetative cells, accounting for 0.07% of the total population, we calculated the effects of contaminating metabolites from *B. subtilis* S4ga on the metabolites of the ADI pathway in *E. faecalis* T6a1. Based on the results of the intracellular metabolomic ana­lysis of the *B. subtilis* S4ga incubation alone, the amounts of arginine, citrulline, and ornithine in vegetative *B. subtilis* S4ga corresponded to 0.08–2% of each metabolite of co-incubated* E. faecalis* T6a1 (Table S3). Therefore, even if the intracellular metabolites of *E. faecalis* T6a1 had been contaminated by vegetative *B. subtilis* S4ga cells, contamination levels were too small to affect the metabolites of the ADI pathway in *E. faecalis*.

Few studies have shown that non-dye-decolorizing bacteria affect dye decolorization when they are present as minor populations (*e.g*., approximately 1% or less of the total population). From a practical viewpoint, the addition and/or maintenance of *B. subtilis* S4ga at 1% may be a useful index for biological textile wastewater treatments to enhance dye-decolorizing bacteria as well as for probiotics in the gut microbiota to promote beneficial bacteria, such as *E. faecalis* (as a probiotic promoter). Therefore, the present results provide important insights into the roles of minority bacterial populations as key players in dye decolorization and other biological processes.

**Fig. 8.**
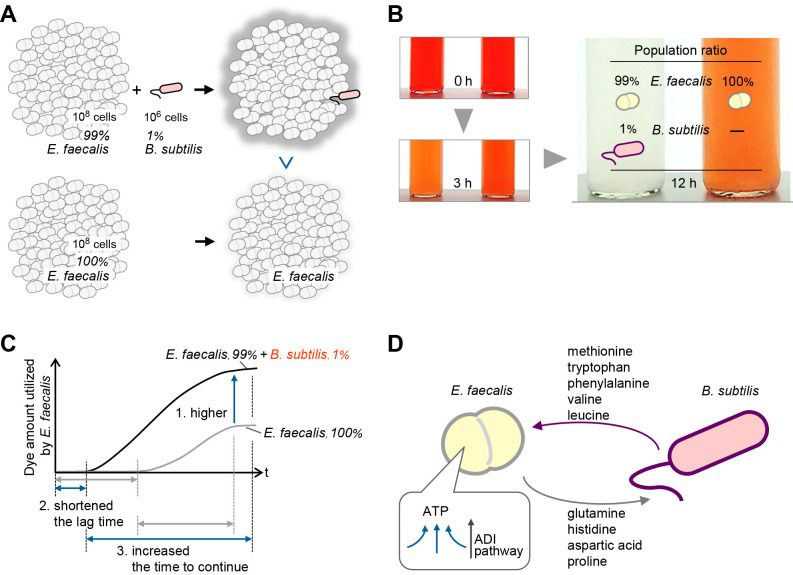
Effects of *Bacillus subtilis* S4ga on the activity of *Enterococcus faecalis* T6a1. (A) Schematic illustration of a 1% population. (B) Schematic photographs of the dye decolorization assay showing a higher decolorization rate with the co-incubation. (C) Schematic depiction showing the enhancement of dye-decolorizing activity by *B. subtilis*. (D) Schematic illustration showing the cross-feeding interaction of amino acids between *E. faecalis* and *B. subtilis*.

In conclusion, we herein demonstrated that *B. subtilis* S4ga enhanced the activity of *E. faecalis* T6a1, even when it accounted for only 0.4% of the total population, as demonstrated by the results of the dye decolorization assay using non-dye-decolorizing *B. subtilis* S4ga and dye-decolorizing *E. faecalis* T6a1. The enhancing effects of *B. subtilis* S4ga on the activity of *E. faecalis* T6a1 were observed not only as a high maximum dye decolorization rate, but also as a shorter lag time and longer active time of dye decolorization, and the amount of dye decolorization was doubled. During the co-incubation, *B. subtilis* S4ga reduced the ORP value to approximately 0 mV by consuming oxygen and/or producing reducing agents, such as amino acids. The amino acids produced by *B. subtilis* S4ga were the same as those consumed by *E. faecalis* T6a1, whereas the amino acids produced by *E. faecalis* T6a1 were the same as those consumed by *B. subtilis* S4ga. In other words, these strains have a mutual supply relationship for specific amino acids. This may have increased the intracellular ATP content of *E. faecalis* T6a1 and *B. subtilis* S4ga, partly via the activation of the ADI pathway of *E. faecalis* T6a1. These results are summarized in Fig. 8. The present study provides novel insights into co-existing minor populations in microbial communities as well as a more detailed understanding of bacterial control. Further studies are needed to clarify whether the combination of these species or the presence of *B. subtilis* is important for enhancing the whole-cell activity of co-existing bacteria.

## Citation

Yamanashi, Y., and Ito, T. (2022) A Minority Population of Non-dye-decolorizing *Bacillus subtilis* enhances the Azo Dye-decolorizing Activity of *Enterococcus faecalis*. *Microbes Environ ***37**: ME21080.

https://doi.org/10.1264/jsme2.ME21080

## Supplementary Material

Supplementary Material
