# Supplementary material for: A Minority Population of Non-dye-decolorizing Bacillus subtilis enhances the Azo Dye-decolorizing Activity of Enterococcus faecalis
Source: Microbes Environ. 2022 May 31;37(2):ME21080. doi: 10.1264/jsme2.ME21080 (PMC9530732; doi:10.1264/jsme2.ME21080)
Supplement: Supplementary file 1 — Supplementary Material [file 37_21080_s1.pdf]

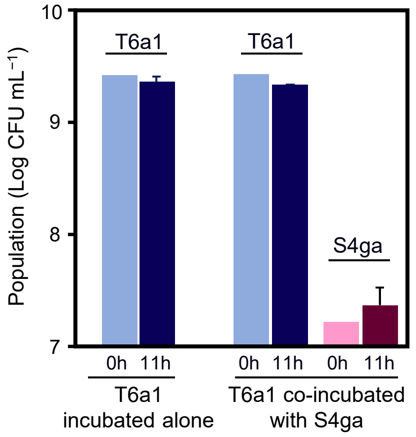

Figure S1. Populations of *E. faecalis* T6a1 incubated alone or co-incubated *E. faecalis* T6a1 and *B. subtilis* S4ga before or after the incubation for 11 h. The initial Congo red dye concentration was 60 mg L^{-1}.

Figure S1. Yamanashi & Ito

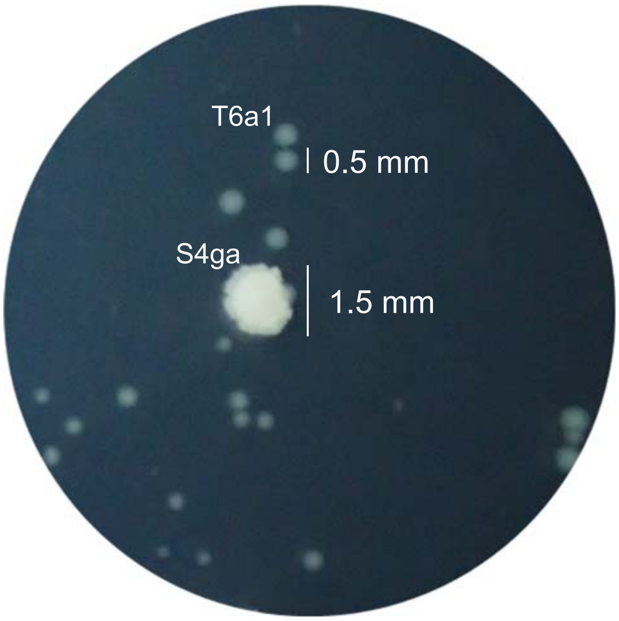

Figure S2. *E. faecalis* T6a1 colonies (small circular) appeared with a *B. subtilis* S4ga colony (large irregular) on a 10% LB agar plate incubated for 12 h.

Figure S2. Yamanashi & Ito

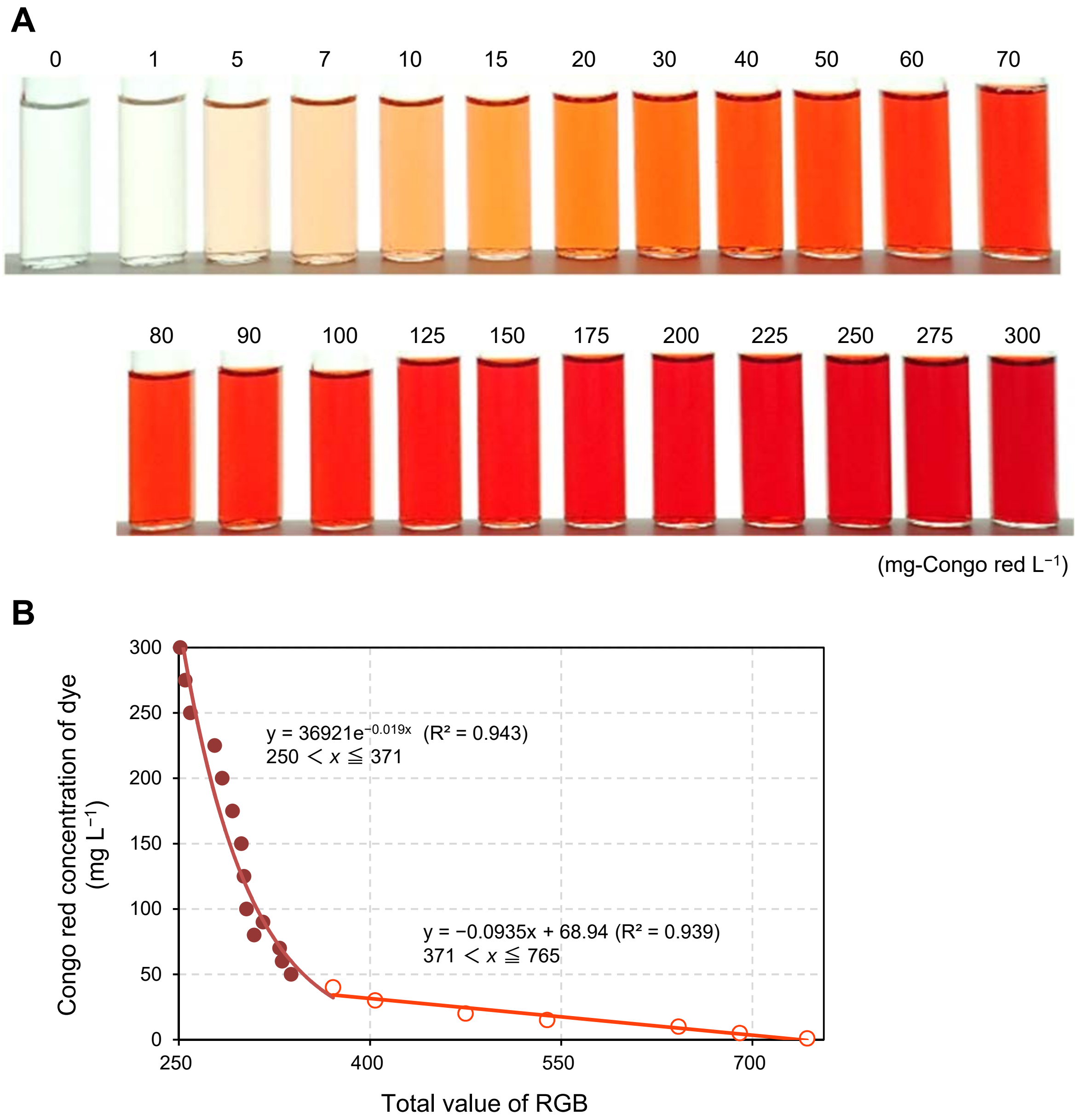

Figure S3. Dye concentration estimations by measuring RGB values in images of incubating tubes. (A) Images of standard solutions of Congo red dye in 10% LB medium at concentrations ranging between 0 and 300 mg L^{-1}. (B) Calibration curves of the total RGB value versus Congo red dye concentrations; a linear approximation for concentrations <40 mg L^{-1} and an exponential approximation for concentrations between 40 and 300 mg L^{-1}.

Figure S3. Yamanashi & Ito

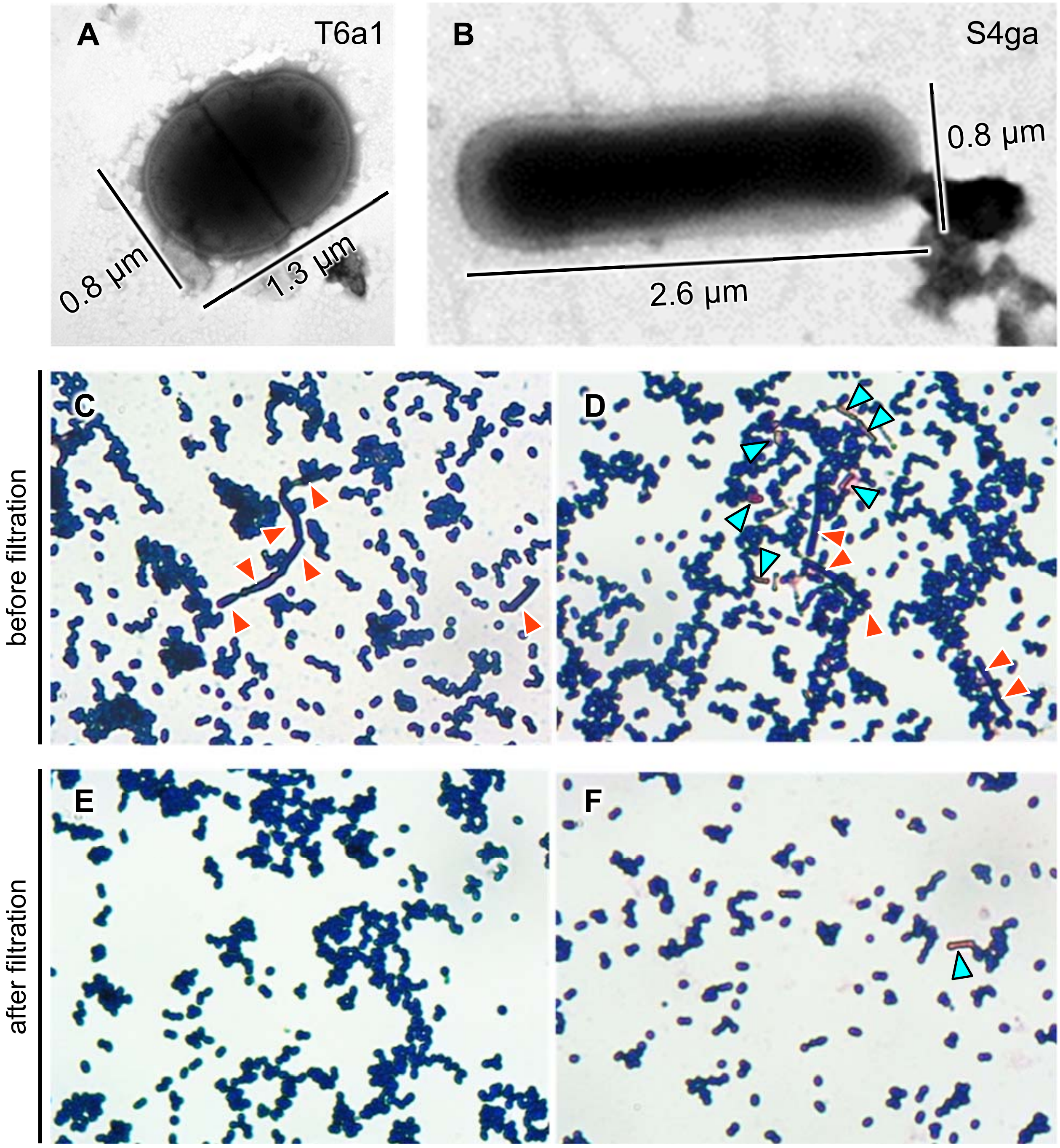

Figure S4. Removal of non-dye decolorizing *B. subtilis* S4ga cells from the co-incubation solution. Electron micrographs of *E. faecalis* T6a1 (A) and *B. subtilis* S4ga (B) when negatively stained with 2% (w/v) phosphotungstic acid and observed under a transmission electron microscope (JEM-1010; JEOL, Tokyo, Japan) at an acceleration voltage of 80 kV. Microscopic images of gram-stained cell suspension of the co-incubation of *E. faecalis* T6a1 and *B. subtilis* S4ga, before (C, D) and after (E, F) 0.8-µm filtration. Both strains were gram-positive (colored blue-violet). Among *B. subtilis* S4ga cells, vegetative cells are indicated with red arrows, whereas endospores and likely nonvegetative thin cells appear pale red (blue arrows).

Figure S4. Yamanashi & Ito

Table S1. List of non-dye-decolorizing and dye-decolorizing bacterial isolates used in this study and their phylogenetic identification.

Strain	Dye decolorization ability	Dye decolorization rate of co-incubation with strain T6a1 (mg L^{-1} h^{-1})[a]	Phylogenetic identification	Sequence similarity (%)	Accession number
S4ga	−	5.3	*Bacillus subtilis*	100	LC557810
S4gb	−	4.9	*Bacillus subtilis*	100	LC557811
C16caA	−	4.3	*Bacillus* sp.	97	LC557812
C47ea	−	3.5	*Bacillus aryabhattai*	99	LC557813
A14d3B	−	3.5	*Bacillus subtilis*	99	LC557814
D3da	−	3.3	*Bacillus* sp.	99	LC557815
C8ba	−	3.3	*Bacillus* sp.	99	LC557816
T6a1	+	3.5[b]	*Enterococcus faecalis*	99	LC557817

+; decolorization of dye
−; not decolorized
[a] incubated in LB medium
[b] strain T6a1 incubated alone

Table S2. Normalized intensities of water-soluble primary metabolites extracted from *E. faecalis* T6a1 during dye decolorization when *E. faecalis* T6a1 was co-incubated with *B. subtilis* S4ga and incubated alone.

Primary metabolite	Normalized intensity				Log2 Fold Change	*p*-value
	T6a1-1	T6a1-2	T6a1 w/ S4ga-1	T6a1 w/ S4ga-2		
Citrulline	0.0056	0.0052	0.0105	0.0114	1.0168	0.007
Adenylsuccinic acid	0.0023	0.0016	0.0059	0.0044	1.4282	0.056
Proline	0.2262	0.2584	0.4423	0.5700	1.0628	0.057
Histidine	0.5971	0.6319	0.9662	1.2381	0.8428	0.071
Ornithine	0.5348	0.5809	0.7050	0.8674	0.4951	0.114
S-Adenosylmethionine	0.0082	0.0082	0.0109	0.0145	0.6375	0.129
Xanthine	0.0007	0.0004	0.0010	0.0008	0.6977	0.139
Alanine	0.0398	0.0405	0.0624	0.0954	0.9734	0.143
Adenine	0.0201	0.0173	0.0310	0.0517	1.1466	0.162
Uridine	0.0013	0.0009	0.0015	0.0020	0.6938	0.167
Aspartic acid	1.0941	1.0808	1.3349	1.8171	0.5353	0.180
Hypoxanthine	0.0019	0.0023	0.0029	0.0048	0.8896	0.212
Oxidized glutathione	0.0036	0.0062	0.0073	0.0073	0.5721	0.215
Arginine	0.0437	0.0560	0.0595	0.0740	0.4207	0.218
Threonine	0.0331	0.0391	0.0470	0.0824	0.8422	0.253
Methionine	0.0101	0.0087	0.0122	0.0286	1.1196	0.311
Guanosine monophosphate	0.0604	0.0592	0.0412	0.0572	0.2808	0.320
NAD	0.0064	0.0069	0.0069	0.0107	0.4073	0.374
Histamine	0.0006	0.0006	0.0009	0.0014	0.9735	0.374
Leucine	0.0941	0.0946	0.1040	0.3002	1.0987	0.387
Phenylalanine	0.0600	0.0707	0.0679	0.2436	1.2522	0.413
Glutamine	0.0350	0.0647	0.0365	0.0383	0.4149	0.490
Isoleucine	0.0640	0.0600	0.0784	0.1882	1.1051	0.492
Tyrosine	0.0134	0.0275	0.0165	0.0454	0.5966	0.582
Glutathione	0.0461	0.1264	0.0472	0.0834	0.4015	0.681
5-Glutamylcysteine	0.0751	0.1137	0.0800	0.1372	0.2023	0.720
Adenosine	0.2702	0.1370	0.1535	0.2050	0.1839	0.766
Lactic acid	0.0746	0.1223	0.1107	0.0996	0.0948	0.810
Glycine	0.0058	0.0094	0.0073	0.0088	0.0862	0.836
2-Morpholinoethanesulfonic acid[a]	1.0000	1.0000	1.0000	1.0000	0.0000	-
Guanosine	0.0250	0.0179	0.0157	0.0267	0.0163	-
Succinic acid	0.0211	0.0189	0.0202	0.0208	0.0365	-
Niacinamide	0.0022	0.0027	0.0023	0.0029	0.0758	-
Methionine sulfone[a]	1.8281	1.7697	1.8318	1.5801	0.0765	-
Cytidine monophosphate	0.1923	0.1838	0.1580	0.1961	0.0869	-
Serine	0.0619	0.0776	0.0732	0.0763	0.0995	-
Adenosine monophosphate	0.4267	0.4100	0.3933	0.5678	0.1998	-
Acetylcarnitine	0.0013	0.0021	0.0017	0.0024	0.2772	-
Ophthalmic acid	0.0069	0.0088	0.0082	0.0117	0.3454	-
Asparagine	0.0088	0.0151	0.0152	0.0195	0.5437	-
Carnitine	0.0452	0.0383	0.0607	0.0686	0.6321	-
Choline	0.0131	0.0138	0.0160	0.0279	0.7027	-
Cytidine	0.0265	0.0171	0.0214	0.0511	0.7355	-
Methionine sulfoxide	0.0024	0.0021	0.0016	0.0060	0.7488	-
Asymmetric dimethylarginine	0.0024	0.0021	0.0030	0.0046	0.7767	-
Tryptophan	0.0151	0.0158	0.0167	0.0440	0.9742	-
Valine	0.0757	0.0769	0.1069	0.2375	1.1741	-
4-Aminobutyric acid	0.0008	0.0009	0.0014	0.0027	1.3116	-
Carnosine	0.0006	0.0002	0.0007	0.0023	1.8895	-
Cystathionine	0.0017	0.0069	0.0005	0.0016	2.0563	-
Lysine	5.4657[b]	5.4616[b]	6.9557[b]	9.7226[b]	0.6100	0.173
Glutamic acid	2.7319[b]	2.7431[b]	3.3127[b]	4.4373[b]	0.5013	0.180

[a] internal standard added.
[b] saturated value.
The metabolites are arranged in order of *p*-value. At the top of the list are significantly different metabolites between the co-incubated and sole-incubated *E. faecalis* T6a1.
Not detected: 2-Aminobutyric acid, Acetylcholine, Aconitic acid, Allantoin, Argininosuccinic acid, cCMP, Cholic acid, Citicoline, Citric acid, Cysteamine, Cystine, Cytosine, Dopa, Epinephrine, Fumaric acid, Homocysteine, Homocystine, Isocitric acid, Kynurenine, Malic acid, Norepinephrine, Orotic acid, S-Adenosylhomocysteine, Serotonin, Taurocholic acid, Thymine, Uric acid, Cysteine.
Weak peak: cAMP, FAD, Guanine, Dopamine, Thymidine monophosphate, Pyruvic acid, Creatinine, cGMP, Nicotinic acid, Inosine, 4-Hydroxyproline, Thymidine, Creatine, Pantothenic acid, Uracil, FMN.
Not determined due to peak overlap: Dimethylglycine, Symmetric dimethylarginine, 2-Ketoglutaric acid.

Table S3. Simulation of the effects of contaminating metabolites from *B. subtilis* S4ga on the metabolites of the ADI pathway in *E. faecalis* T6a1.

Strain	Population (CFU mL^{-1})	Normalized intensity		
		Arginine	Citrulline	Ornithine
E. faecalis T6a1	1×10^9	0.06675[a]	0.01095[a]	0.07862[a]
B. subtilis S4ga	7×10^5	0.00005[b]	0.00026[b]	0.00006[b]
Ratio	0.07%[c]	0.07%	2.4%	0.08%

[a] values of the intracellular metabolites of *E. faecalis* T6a1 co-incubated with *B. subtilis* S4ga (data from Table S2).

[b] calculated from the results of the intracellular metabolomic analysis of the *B. subtilis* S4ga incubation alone (8×10^8 CFU mL^{-1}).

[c] based on the assumption that the intracellular metabolites of *E. faecalis* T6a1 were contaminated by *vegetative B. subtilis* S4ga cells at the population ratio of 0.07%, which we detected some endospores or non-vegetative cells after three time filtration (Fig. S4E and F).
